# Effect of Functionalized Polyethylene Wax on the Melt Processing and Properties of Highly Filled Magnesium Hydroxide/Linear Low-Density Polyethylene Composites

**DOI:** 10.3390/polym15112575

**Published:** 2023-06-04

**Authors:** Rujie Li, Shiai Xu, Jiajun Xu, Tongtong Pan, Beibei Sun, Li Dang

**Affiliations:** 1 College of Chemical Engineering, Qinghai University, Xining 810016, China; lrj_superman@163.com (R.L.); pantongtong1024@163.com (T.P.); qdhysun@163.com (B.S.); dangli@163.com (L.D.); 2 College of Materials Science and Engineering, East China University of Science and Technology, Shanghai 200237, China; kaju6017@163.com

**Keywords:** highly filled composites, magnesium hydroxide, linear low-density polyethylene, maleic anhydride grafted polyethylene wax, flame-retardant polymer composite

## Abstract

The poor processing and rheological properties of highly filled composites caused by the high loading of fillers can be improved with the use of maleic anhydride grafted polyethylene wax (PEWM) as compatibilizer and lubricant. In this study, two PEWMs with different molecular weights were synthesized by melt grafting, and their compositions and grafting degrees were characterized by Fourier transform infrared (FTIR) spectroscopy and acid-base titration. Subsequently, magnesium hydroxide (MH)/linear low-density polyethylene (LLDPE) composites with 60 wt% of MH were prepared using polyethylene wax (PEW) and PEWM, respectively. The equilibrium torque and melt flow index tests indicate that the processability and fluidity of MH/MAPP/LLDPE composites are significantly improved with the addition of PEWM. The addition of PEWM with a lower molecular weight leads to a substantial reduction in viscosity. The mechanical properties are also increased. The limiting oxygen index (LOI) test and cone calorimeter test (CCT) show that both PEW and PEWM have adverse effects on flame retardancy. This study provides a strategy to simultaneously improve the processability and mechanical properties of highly filled composites.

## 1. Introduction

Linear low-density polyethylene (LLDPE) is a commercially important thermoplastic and is widely used in cables, wires, and pipes due to its high toughness, chemical stability, environmental stress-cracking resistance and thermal properties [1,2,3,4,5]. However, it is highly flammable [1,3,6] and smoke and combustible gases can be generated in large quantities during combustion [7,8,9]. One possible solution to these problems is to add flame-retardant additives to reduce the flammability of LLDPE [7,8,9,10,11], and the most common additives includes metal hydroxides, phosphorus- or nitrogen-containing compounds, and halogen flame retardants [12,13,14,15]. As halogen flame retardants are toxic and potentially harmful to the environment, special attention has been given to metal hydroxides because of their non-toxicity and low cost [11,16,17,18]. Magnesium–hydroxide (MH) and aluminium–hydroxide (ATH) are well-known metal hydroxides. MH has a higher smoke suppression capability and greater thermal stability than ATH [17,19], and it can protect the material through endothermic dehydration and the formation of a heat barrier during combustion [20,21,22,23,24]. However, MH can act as an effective flame-retardant filler only at high concentrations (50–60 wt%) [25,26,27,28], and it also has a negative impact on the mechanical, melt-processing and flow properties of highly filled polymer composites [8,9,19,21,29], because inorganic fillers are likely to agglomerate and are poorly dispersed in the matrix due to incompatibility with the matrix. The compatibility of MH with the polymer matrix can be improved by the surface modification of fillers and the addition of compatibilizers [25,26,27,28]. Noh et al. [30] found that surface modification of MH nanoparticles with hexylphosphoric acid (HPA) led to the formation of a hydrophobic surface and a more uniform dispersion of MH nanoparticles. It is worth noting that 10 wt% of HPA-modified MH showed better thermal stability than 50 wt% of pristine MH, which could be attributed to the uniform dispersion of MH particles. Michael et al. [11] found that LLDPE with a high content (60 wt%) of magnesium–dihydroxide (MDH) showed particle–particle interactions that could be reduced by using maleic-acid-anhydrite-grafted LLDPE (4–5 wt%). Thus, it is important for MH particles to be uniformly dispersed in the polymer matrix. Savas et al. [31] prepared HH/LLDPE composites with 40–60 wt% of HH and compared the effects of compatibilizers and modifiers on the properties of these composites. The results showed that the compatibilizers improved the flammability and tensile strength of the composites, and both stearic acid and silane modification increased the toughness of the composites. However, it was also noted that surface modification is rather complex and that the use of a compatibilizer can deteriorate the processability of the composites [32,33,34]. Therefore, it is important to improve the processing and mechanical properties of highly filled composites in order to expand their industrial applications.

Polyethylene wax (PEW) is an important fine chemical with many outstanding properties, such as low cost, high thermal stability, dispersibility and fluidity [35,36,37]. PEW has good compatibility with LLDPE due to their similar molecular structure and polarity. It is a “small molecule” with a low molecular weight, and its good lubricity contributes to improving the processing performance of MH/LLDPE composites. The molecular weight of PEW has an effect on its melting point and viscosity, while the properties of PEW as an additive can affect the properties of the composites. PEW grafted with maleic anhydride (MAH) can improve its properties and thus broaden its applications. The introduction of the polar groups of MAH in PEW increases its compatibility with MH, which is conducive to improving the dispersion of MH and reducing the formation of agglomerates [35].

The main objective of this study is to solve the problem of low flowability and poor of mechanical properties due to high loading of fillers. To the best of our knowledge, there has been no study examining the effect of PEW or PEWM on the properties of highly filled MH/LLDPE composites.

**Figure 1:**
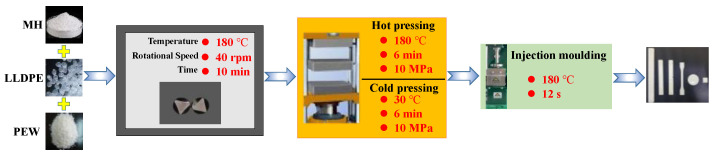
Schematic diagram of the preparation of highly filled MH/LLDPE composites.

The schematic diagram of the preparation of highly filled MH/LLDPE composite is shown in Figure 1. The highly filled MH/LLDPE composites with 60 wt% of MH were prepared to analyze the effects of molecular weights of PEW and PEWM in MH/LLDPE composites on the processability, fluidity, mechanical properties, thermal stability and flame retardancy of highly filled MH/LLDPE composites.

## 2. Materials and Methods

### 2.1. Materials

MH (molecular weight = 58.32, density = 2.36 g/cm^3^, analytically pure) was supplied by Tianjin Damao Chemical Reagent Factory (Tianjin, China). LLDPE 7042 (density = 0.918 g/cm^3^) was supplied by Shanghai Kaibo Species Cable Material Factory Co., Ltd. (Shanghai, China). Two polyethylene waxes (PEW1, TLZJ-5Y, viscosity-average molecular weight (Mυ) = 1700, melting point = 110 °C, density = 0.920 g/cm^3^, acidity = 0; PEW2, TLZJ-1, Mυ =4500, melting point = 115 °C, density = 0.930 g/cm^3^, acidity = 0) were supplied by Chengdu Tongli Auxiliaries Co., Ltd. (Chengdu, China). MAH (relative density = 1.48 g/cm^3^, melting point = 52.8 °C, boiling point = 202.2 °C, analytically pure) was supplied by Xiya Chemical Technology Co., Ltd. (Shandong, China). Benzoyl peroxide (BPO, melting point = 105 °C, molecular weight = 242.23, analytically pure) was supplied by Shanghai Macklin Biochemical Technology Co., Ltd. (Shanghai, China).

### 2.2. Preparation and Purification of PEWM

**Figure 2:**
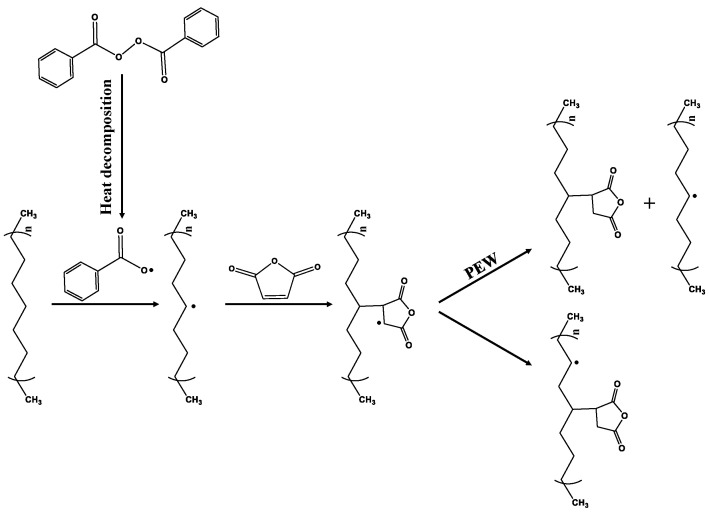
Synthesis reaction of PEWM.

PEWM was prepared by melt blending, where the PEW/MAH/BPO ratio was set at 100/7/0.5, which is schematically shown in Figure 2. PEW and MAH were put into a three-necked round-bottom flask and then heated and mixed thoroughly by stirring. After that, the initiator BPO was added and reacted for 1 h. The crude PEWM obtained was dissolved in hot xylene, and then quickly poured into a large amount of acetone to obtain flocculent precipitate. Finally, purified PEWM was obtained after vacuum filtration suction. The product was dried under vacuum at 120 °C for 12 h before use and analysis.

### 2.3. Preparation of MH/LLDPE Composites

MH/LLDPE composites were prepared by the melt-blending method, where the MH content was fixed at 60 wt%, and the polyethylene wax content was set at 1 wt%, 3 wt% and 5 wt%, respectively. The mixture was added into a Haake torque rheometer at 180 °C and 40 rpm for 10 min to prepare MH/LLDPE composites. For comparison, an MH/LLDPE composite without any polyethylene wax additives was also prepared under the same conditions. The formulations and names are shown in Table 1.

### 2.4. Characterization

The Fourier transform infrared (FTIR) spectra were recorded on the AVATAR 360 FTIR spectrometer (Thermo Nicolet Corp., Waltham, MA, USA) at a scan number of 16 and a resolution of 4 cm^−1^. The grafting degree of MAH of PEW was determined by an acid-base titration method [38].

The equilibrium torque was determined by a torque rheometer (Haake PolyLab QC, Waltham, MA, USA) at 180 °C and 40 rpm for 10 min.

The MFI of compatibilizers was tested at 230°C with a load of 2.16 kg using a melt index tester (MI-4, Gottfertg, Germany) in accordance with ASTM D-1238.

The dynamic rheological properties were measured by a rotational rheometer (Thermo Scientific Mars40, Waltham, MA, USA) in a dynamic frequency sweep from 0.01 to 628 rad/s at a strain of 1% and a temperature of 180 °C. The diameter of the disk was 25 mm, and the thickness was 1.5 mm.

The surface morphology was observed by a JSM-6610LV field emission scanning electron microscope (FE-SEM, Tokyo, Japan). Prior to SEM observation, all samples were fractured in liquid nitrogen and coated with a thin layer of gold.

The tensile properties were measured by a microcomputer-controlled electronic universal testing machine (104B-EX, Wance Test Equipment Co., Ltd., Shenzhen, China) according to GB/T 1040.2-2006, where the clamp distance was 58 mm, the sample thickness was 2 mm, and the stretching speed was 1 mm/min. The average of six specimens was reported. The impact properties were measured by an impact tester (501 J-4, Wance Test Equipment Co., Ltd., Shenzhen, China) according to GB/T 1843-2008 with a notched specimen of 80 × 10 × 4 mm^3^. The average of six specimens was reported.

The limit oxygen index (LOI) was tested by an oxygen index instrument (JF-6, Jionglei Instrument Equipment Co., Ltd., Nanjing, China) according to GB/T 2406-1993 with a specimen of 80 × 10 × 4 mm^3^. The average of six specimens was reported.

Cone calorimeter test (CCT) was tested by a cone calorimeter (CCT, Modisco Combustion Technology Instrument Co., Ltd., Kunshan, China) at a heat flux of 35 kW/m^2^ according to ISO 5660 with a specimen of 100 × 100 × 3 mm^3^.

## 3. Results and Discussion

### 3.1. Characterization of Polyethylene Wax Additives

**Figure 3:**
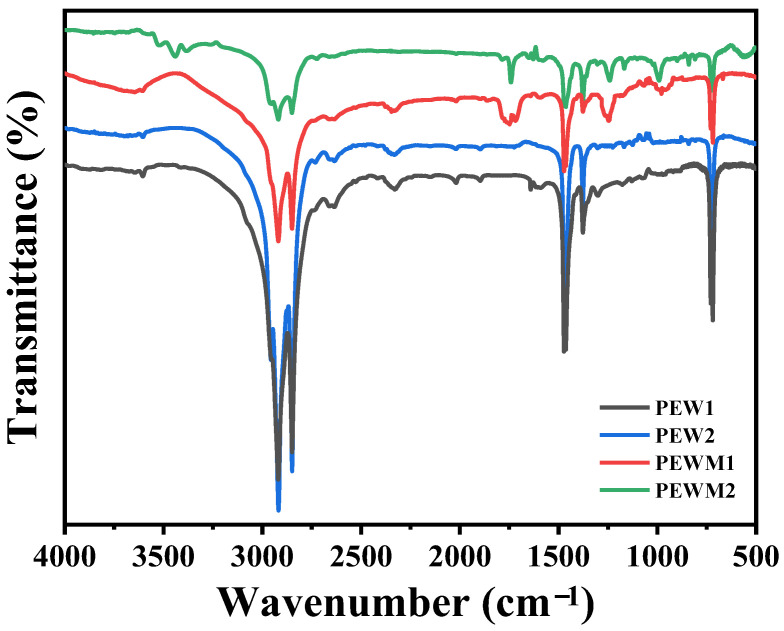
FTIR spectra of PEW and PEWM.

Figure 3 shows the synthesis route and FTIR spectra of polyethylene wax before and after grafting. All samples exhibit absorption peaks at 2919 and 2849 cm^−1^, corresponding to CH_2_ stretching and absorption peaks at 1463 and 729 cm^−1^ corresponding to the bending and rocking vibrations of CH_2_, respectively [35,39]. Compared to PEW, PEWM shows three absorption peaks at 1781 cm^−1^, 1226 cm^−1^ and 955 cm^−1^, which correspond to the stretching vibration of cyclic anhydride and the stretching and bending vibration of COC, respectively [35]. These results show that the MAH has been successfully grafted on the molecular chain of polyethylene wax. The grafting degree of PEWM1 and PEWM2 is 3.62% and 3.60%, respectively.

### 3.2. Torque Analysis

**Figure 4:**
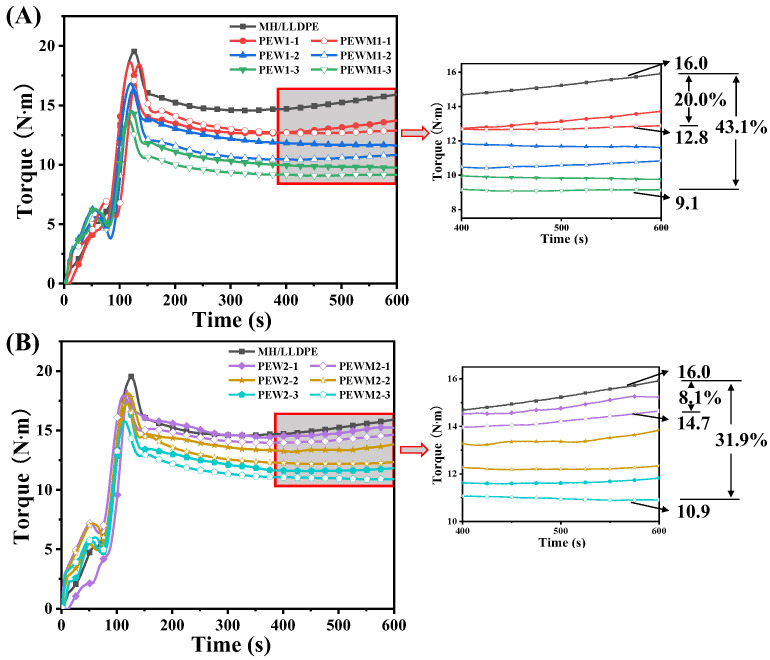
The torque versus time curves for: (**A**) MH/LLDPE composites with PEW1 and PEWM1; and (**B**) MH/LLDPE composites with PEW2 and PEWM2.

Figure 4 shows the effects of polyethylene wax additives on the processability of the composites. The torque of all samples reaches equilibrium within the prescribed mixing time. The equilibrium torque value of the MH/LLDPE composite is approximately 16.0 N·m. The torque values of highly filled composites are reduced due to the addition of PEW or PEWM. The torque value of PEW1-1, PEWM1-1, PEW2-1 and PEWM2-1 is 13.7 N·m, 12.8 N·m, 15.2 N·m and 14.7 N·m, which is decreased by 14%, 20%, 5% and 8% compared to MH/LLDPE, respectively. This is because the molecular weight of the polyethylene wax additive is smaller than that of LLDPE, which can reduce the interaction between polymers [40]. The presence of PEW or PEWM has a positive effect on the processability, which is more pronounced at higher concentrations or smaller molecular weights. Given the same loading of PEW and PEWM, the MH/PEWM/LLDPE composites have lower equilibrium-torque values than MH/PEW/LLDPE composites. This is because PEWM can react with MH due to the presence of MAH [41], and it has better filler wettability and can reduce the formation of filler agglomerates. PEWM1-3 exhibits excellent processability, and the equilibrium torque value was reduced by 43%. These results imply that the molecular weights and functional groups of polyethylene wax additives play important roles in improving the processability of the highly filled composites.

### 3.3. Morphology

**Figure 5:**
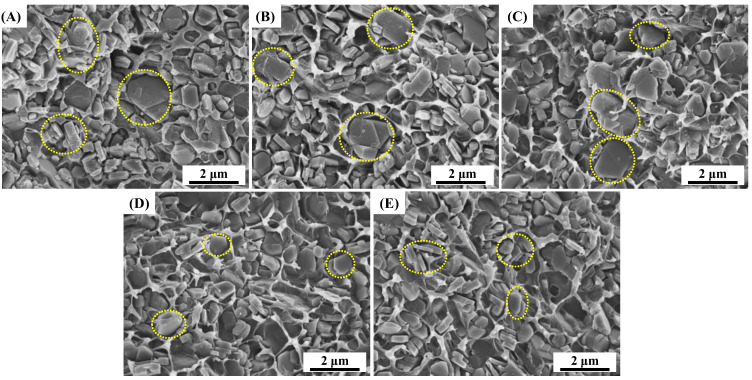
SEM images of the cryo-fractured surfaces of MH/LLDPE, MH/PEW /LLDPE and MH/PEWM/LLDPE composites: (**A**) MH/LLDPE; (**B**) PEW1-1; (**C**) PEW2-1; (**D**) PEWM1-1; and (**E**) PEWM2-1.

The effects of PEW and PEWM on the dispersion of fillers in the matrix and the compatibility between them in MH/LLDPE composites were determined by SEM. As shown in Figure 5A, the addition of a large number of MH particles into the LDDPE matrix results in the formation of many voids between the particles and the matrix and a large number of agglomerates in the MH/LLDPE composite (marked by yellow circles), which is attributed to the poor compatibility between them and the increased contact between particles. Figure 5B,C shows the presence of more agglomerates (marked by yellow circles) in the MH/PEW/LLDPE composites compared to the MH/LLDPE composites. The compatibility of the particle with the matrix is not improved with the addition of PEW because PEW is a non-polar structure like LLDPE, and it is poorly compatible with polar MH particles. Polymer melt with low viscosity results in lower shear during processing, which can promote the formation of agglomerates [29]. Notably, the small agglomerate size in the yellow circle in Figure 5D,E indicates that the addition of PEWM improves the dispersion of MH particles in the LLDPE matrix. This can be attributed to the reaction of MAH groups in the PEWM with the reactive hydroxyl groups on the surface of the MH particles, which makes MH more wettable by the polymer matrix. It is also difficult for wetted MH particles to contact with other MH particles to form agglomerates [40].

### 3.4. MFI

**Figure 6:**
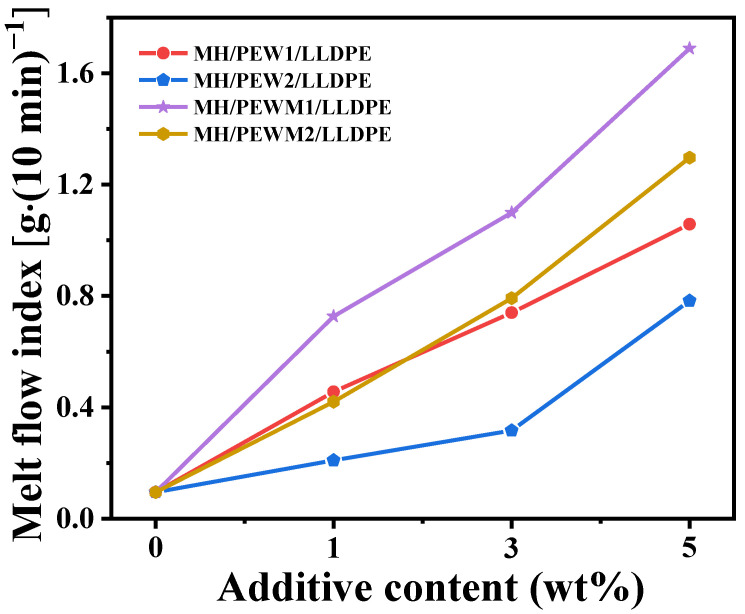
The curves of the melt-flow index versus the content of the polyethylene wax additive.

The MFI test is commonly used to test the fluidity of composites and it provides a visual representation of the flow of composites. As shown in Figure 6, the MH/LLDPE composite exhibits the smallest MFI value (0.10 g/10 min). The MFI value of MH/PEWM/LLDPE composites with 1 wt% of PEWM1 is increased by 667%. The fluidity of the highly filled MH/LLDPE composites is significantly improved with the addition of PEW and PEWM, and the higher the content, the better the fluidity of the composite. This can be attributed to the lubrication of PEW and PEWM, which can reduce filler−polymer and polymer−polymer interactions and thus increase the mobility of the macromolecular chain. Compared to the MH/PEW2/LLDPE and MH/PEWM2/LLDPE composites, the MH/PEW1/LLDPE and MH/PEWM1/LLDPE composites exhibit better fluidity due to the lower molecular weight and better lubrication of PEW1 and PEWM1. MH/PEWM/LLDPE composites have lower MFI values than MH/PEW/LLDPE composites, which can be attributed to the reduction of MH aggregates and the improved dispersion of MH particles.

### 3.5. Dynamic Rheological Properties

**Figure 7:**
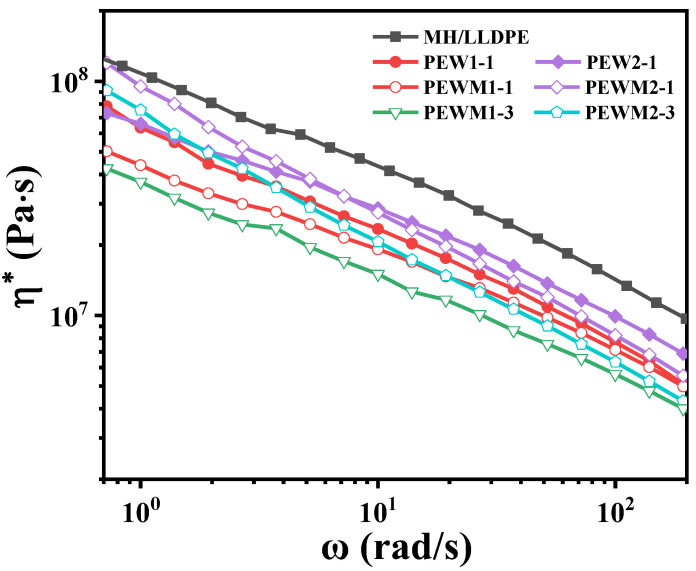
The curve of complex viscosity (η*) versus frequency (*ω*) for composites.

Rheological analysis is an important means of characterizing the microstructure of composites because the viscoelastic response is related not only to the short-range structure of fillers but also to the long−range interaction between fillers and matrix [41,42,43]. Figure 7 shows the η* vs. *ω* curves for MH/LLDPE, MH/PEW/LLDPE and MH/PEWM/LLDPE composites.

The viscosity behaviors of all the samples are characteristic of non-Newtonian pseudoplastic liquids, which exhibit typical shear-thinning behavior (i.e., η* decreases with the increase of *ω*). Clearly, the MH/LLDPE composites exhibit high viscosity, and the melt viscosity decreases with the addition of PEW and PEWM. Compared to PEW, PEWM can significantly reduce the viscosity due to the ability of MAH groups to reduce the formation of MH agglomerates. This is consistent with the SEM results (Figure 5). It is also found that the smaller the molecular weight of the polyethylene wax additive, the lower the complex viscosity of the sample. This is because PEW and PEWM have lower molecular weights than LLDPE, which reduces the density of molecular-chain entanglement and enhances the movement of molecular chains. Thus, MAH-grafted PEW is more effective in reducing the melt viscosity of highly filled MH/LLDPE composites, which is consistent with the results in Figure 4.

**Figure 8:**
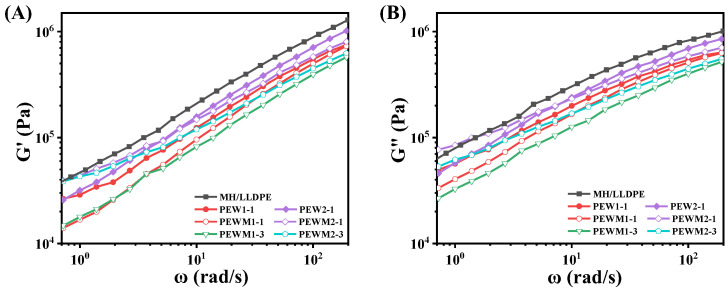
(**A**) Storage modulus (*G*’); and (**B**) loss modulus (*G*”) versus frequency (*ω*) curves for composites.

Figure 8A,B shows the curves of the storage modulus and loss modulus as a function of frequency for MH/LLDPE, MH/PEW/LLDPE and MH/PEWM/LLDPE composites, respectively. No typical termination behaviors are observed in all samples (*G*’ ∝ *ω*^2^, *G*” ∝ *ω*), because the relaxation process occurs at lower frequencies and takes a longer time due to the high loading of the fillers [33]. The longer relaxation time is related to the filler–filler and filler–polymer interactions [40,43]. In comparison to MH/LLDPE, *G*’ and *G*” are significantly decreased with the addition of PEW or PEWM. This may be because PEW and PEWM have lower molecular weights compared to LLDPE, and resulting the composites exhibit a more solid-like response. At the same content of PEW and PEWM, the MH/PEWM/LLDPE composites have lower *G*’ and *G*” values than the MH/PEW/LLDPE composites, which is attributed to the improved dispersion of the MH particles in the LLDPE matrix and the reduced interactions between fillers. Similar results have also been reported previously [32].

### 3.6. Mechanical Properties

**Figure 9:**
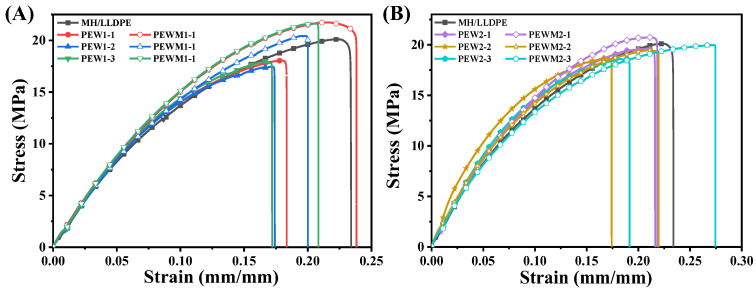
Stress–strain curves for: (**A**) MH/LLDPE composites with PEW1 and PEWM1; and (**B**) MH/LLDPE composites with PEW2 and PEWM2, respectively.

Figure 9 shows the tensile stress–strain curves of all samples, and the data are summarized in Table 2. All samples exhibit brittle fracture and their elongation at break is much lower than 100% [44], which is attributed to the stress-concentration effect of the fillers and the presence of more voids and other defects of phase geometry in the composites. The tensile strength and elongation at break of the MH/LLDPE composites are 20.03 MPa and 23.42%, respectively. Obviously, the addition of PEW leads to a reduction in the tensile strength and elongation at break to different extents. This is because the filler and the matrix are physically bonded and the affinity between them could not be enhanced with the addition of PEW due to the similar non–polar structure of PEW and LLDPE [40]. The addition of PEW, which has a lower molecular weight compared to LLDPE, reduces the frictional resistance between the components of the composite, making it easier for the components to flow under stress and thus leading to earlier fractures of the composite. At the same loading of PEW, MH/PEW1/LLDPE composites have lower tensile strength and elongation at break than the MH/PEW2/LLDPE composites. A possible explanation is that PEW2 has a stronger intermolecular attraction than PEW1 because of its higher molecular weight [44,45,46,47]. It was found that the tensile properties of the composite are related to the molecular weight of PEW [46]. Figure 9 and Table 2 show that the tensile properties of the MH/PEWM/LLDPE composites are improved compared to MH/PEW/LLDPE composites, indicating that PEWM has a reinforcing effect. PEWM1-1 exhibits excellent tensile properties, and the tensile strength (21.29 MPa) and elongation at break (24.00%) are increased by 6.3% and 2.5% compared to the MH/LLDPE composite, respectively. The reason is that the polar MAH group of PEWM can react with the active hydroxyl group on the surface of MH particles, which improves the interfacial strength between the MH particles and the LLDPE matrix, and thus results in a more efficient transfer of stress [45,46,47]. Interestingly, the tensile strength of the MH/LLDPE composites is decreased from 20.03 MPa to 19.13 MPa, but the elongation at break is increased from 23.42% to 25.80% as the loading of PEWM2 is increased from 0% to 5% due to the lubrication or plasticization effect of PEWM2. Similar findings have been reported for wood–plastics composites [48,49,50].

The Turcsányi empirical equation is often used to quantify the interfacial interaction between the filler and the matrix [44].

(1) $$\ln\left(\frac{\sigma_{yc}}{\sigma_{ym}}\right)+\ln\left(\frac{1+2.5\phi_{f}}{1-\phi_{f}}\right)=B\phi_{f}$$

where *σ_yc_* is the yield strength of the composite, *σ_ym_* is the yield strength of the matrix, *ϕ_f_* is the volume fraction of the filler, and *B* is the strength of the interfacial interaction.

This equation can be used for binary composites of filler–reinforced polymers, such as calcium–carbonate–reinforced polypropylene and hydrotalcite–filled low–density polyethylene. However, the ternary composite in this work that consists of magnesium hydroxide particles, polyethylene wax and LLDPE, may not be accurately characterized. The Turcsányi equation is extended by Dang et al. for the ternary composites [44]:

(2) $$\ln\left(\frac{\sigma_{yc}}{\sigma_{ym}}\right)+\ln\left(\frac{1+2.5\Phi \left(R\right)}{1-\Phi \left(R\right)}\right)=B\Phi \left(R\right)$$

Here, the polyethylene wax additive is analyzed as another part of the filler, *R* is the mass ratio of the filler to the polyethylene wax additive, and *Φ(R)* is the total volume fraction of the filler and the polyethylene wax additive. Then, Equation (2) is converted into:

(3) $$\left[\ln\left(\frac{\sigma_{yc}}{\sigma_{ym}}\right)+\ln\left(\frac{1+2.5\Phi \left(R\right)}{1-\Phi \left(R\right)}\right)\right]R/\Phi \left(R\right)=BR$$

**Figure 10:**
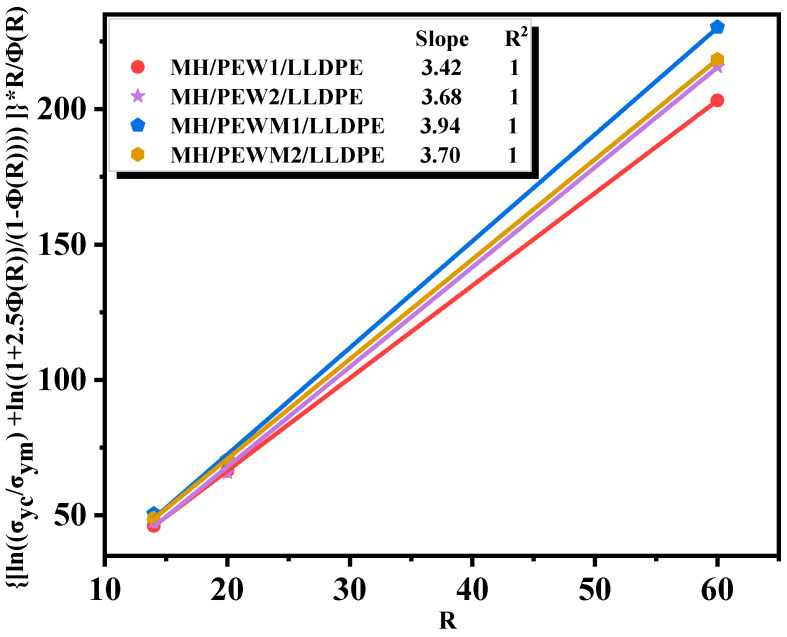
Fitted plots of the extended Turcsányi equation for composites.

The graph is plotted with $\left\{\left[\ln\left(\sigma_{yc}/\sigma_{ym}\right)+\ln\left(\left(1+2.5\Phi \left(R\right)\right)/\left(1-\Phi \left(R\right)\right)\right)\right]\right\}*R/\Phi \left(R\right)$ as the vertical coordinate and *R* as the horizontal coordinate, and the *B* value is obtained by fitting. As shown in Figure 10, it is found that *B* (MH/PEW1/LLDPE) < *B* (MH/PEW2/LLDPE) because of the better physical entanglement of PEW2 with the LLDPE matrix. *B* (MH/PEWM/LLDPE) > *B* (MH/PEW/LLDPE), indicating that PEWM can enhance the interfacial interaction between MH particles and LLDPE matrix. This provides strong support for the previous analysis.

**Figure 11:**
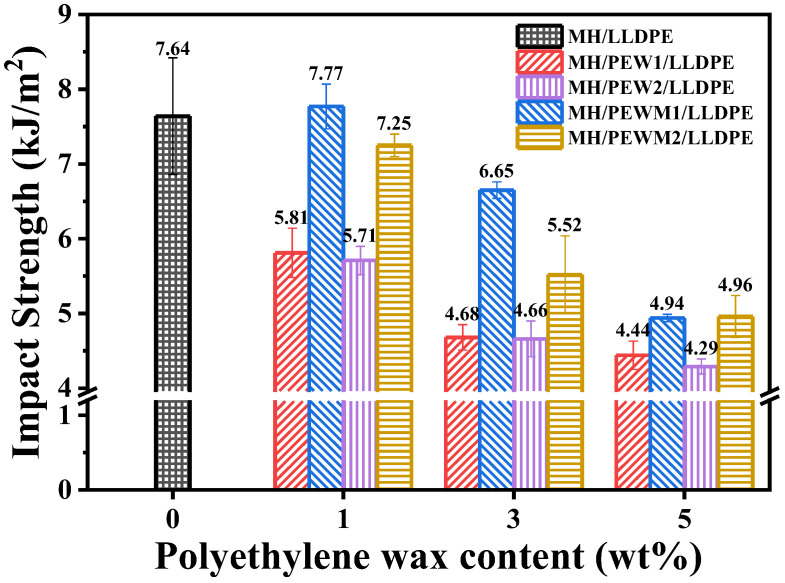
Effect of the type and content of polyethylene wax additives on the impact strength of MH/LLDPE composites.

As shown in Figure 11, the highest impact strength of MH/LLDPE composite is obtained in the MH/LLDPE composite with 1 wt% of PEWM1, which is attributed to the improved dispersion of the MH particles. The impact strength of the MH/PEWM/LLDPE composite is larger than that of the MH/PEW/LLDPE composite at the same loading of PEW and PEWM. Thus, PEWM is effective in improving the interfacial–adhesion strength between particles and the polymer matrix, which makes it possible to absorb more energy. It is also found that the impact strength of the composites decreases with the increasing molecular weight of PEW and PEWM, which is attributed to the better mobility of PEW1 and PEWM1 [48,51]. The impact strength of all samples except PEWM1-1 is less than that of MH/LLDPE and increasing the concentration of PEW and PEWM can reduce the impact strength of MH/LLDPE composites due to its lower molecular weight. Velmurugan et al. [46] also shows that the low–molecular–weight lubricant reduced the efficiency of compatibilizer and consequently the mechanical properties of the composites.

### 3.7. Thermal Stability

**Figure 12:**
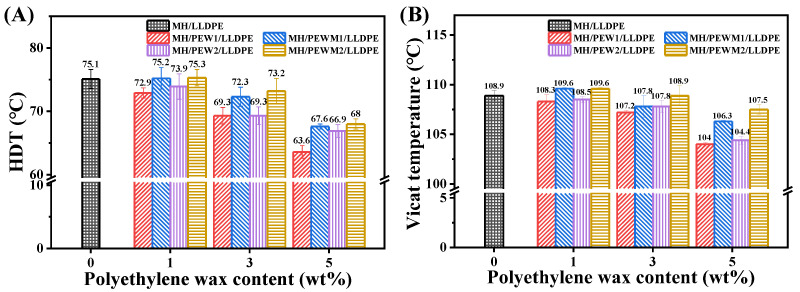
(**A**) HDT; and (**B**) Vicat softening temperature of MH/LLDPE composites with and without polyethylene wax additives.

As shown in Figure 12, the HDT and Vicat softening temperature of MH/LLDPE composites are 75.1 °C and 108.9 °C, respectively, and the addition of PEW1 or PEW2 to the MH/LLDPE composite results in a decrease in HDT and Vicat softening temperature, which is especially noticeable at higher concentrations and smaller molecular weights. This may be due to the reduction of polymer–polymer and polymer–filler interactions with the addition of lower molecular weight polymers [40,52]. Compared with the MH/PEW/LLDPE composites, MH/PEWM/LLDPE composites have a higher HDT and Vicat softening temperature. The HDT and Vicat softening temperature of the MH/PEWM1/LLDPE composites are decreased with the increase of PEWM1 content. The HDT and Vicat softening temperature of PEWM1-1 are 75.2 °C and 109.6 °C, which represent an increase of 0.13% and 0.64% compared to the MH/LLDPE composite, respectively. Similar results are observed for the MH/PEWM2/LLDPE composites. This is because the addition of PEWM increases the polymer–particle interactions and the dispersion of MH particles in the matrix. Similar results are also reported by Dai et al. [50] and Bikiaris et al. [42].

**Figure 13:**
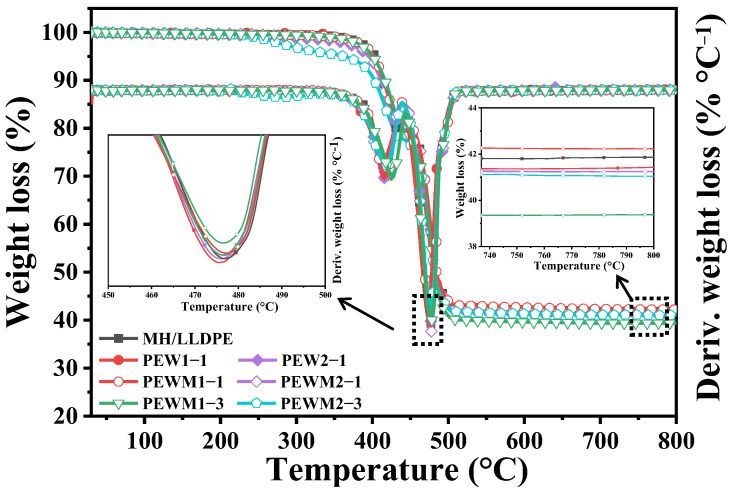
TGA and DTG curves for composites.

The thermal decomposition of MH/LLDPE, MH/PEW/LLDPE and MH/PEWM/LLDPE composites is shown in Figure 13. It is seen that all samples exhibit two weight-loss stages, which is characteristic of immiscible blends whose constituents have different degradation temperatures. The first stage is mainly attributed to the decomposition of the wax and LLDPE matrix, and the second stage is mainly attributed to the thermal decomposition of the MH into magnesium oxide and water. The characteristic parameters, such as the initial decomposition temperature (T_5%_) and maximum decomposition temperature (T_max_), are summarized in Table 3.

The T_5%_ and T_max_ values of the MH/LLDPE composite are 403.1 °C and 477.0 °C, respectively. As shown in Figure 13 and Table 3, the values of T_5%_ and T_max_ decrease slightly with the addition of PEW or PEWM because their thermal stability is lower than that of any polymers [35,36,37]. However, the values of T_5%_ and T_max_ are slightly increased for PEWM1-1, which can be attributed to the improved dispersion of the MH particles by the PEWM1. The T_5%_ and T_max_ values of MH/PEWM1/LLDPE composites are decreased to different extents with the increase of PEWM1. A similar trend is observed for the MH/PEWM2/LLDPE composites. As reported by Mochane et al. [37], the short-chain portion of the wax, as well as the fragments formed by chain cleavage, will have enough energy to leave the matrix at lower temperatures. Therefore, the introduction of more low-molecular-weight waxes will decrease the initial decomposition temperature. In addition, the char yield of PEWM1-1 is higher than that of MH/LLDPE, which also indicates that the interfacial bond between MH particles and LLDPE matrix is enhanced by PEWM1.

### 3.8. Flammability

The cone calorimeter test (CCT) can be used to simulate the real combustion of composites [53]. Here, the flammability of MH/LLDPE, MH/PEW/LLDPE and MH/PEWM/LLDPE composites is assessed by the heat release rate (HRR) and total-heat release (THR), and the smoke-emission ability is evaluated by the smoke–production rate (SPR) and total smoke produced (TSP). Some other parameters, such as ignition time (T_ign_), fire-performance index (FPI) and the average mass loss rate (AMLR), are summarized in Table 4.

**Figure 14:**
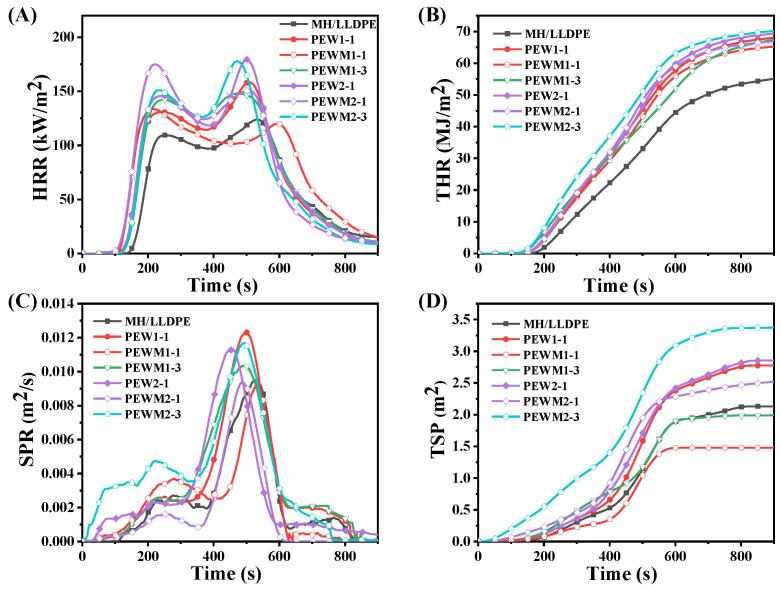
HRR (**A**); THR (**B**); SPR (**C**); and TSP (**D**) for composites.

As shown in Figure 14A,B and Table 4, the peak heat-release rate (PHRR) and THR values of the MH/LLDPE composites are 123.75 kW/m^2^ and 58.43 MJ/m^2^, respectively. The PHRR and THR values of the composite decrease to different extents with the addition of PEW and PEWM. The MH/LLDPE composite exhibits the longest ignition time and the highest FPI, indicating that PEW and PEWM reduce the flame retardancy of MH/LLDPE composites. This is probably because PEW and PEWM burn easily because of the hydrocarbon structure. It is also noted that they are more likely to combust in the early stage because of their low melting points, and as a result more heat is released to promote the combustion of the composite [31,53]. Compared to MH/PEW/LLDPE composites, MH/PEWM/LLDPE composites have lower PHRR and THR values but higher FPI values, which may be related to the improved dispersion of MH particles. The MH/PEWM1/LLDPE composites also show better flame retardancy than MH/PEWM2/LLDPE composites at the same PEWM content, because PEWM1 can enhance interfacial adhesion and thus prevent the release of heat [26,31,53,54]. The LOI test is also performed to evaluate the flame retardancy of MH/LLDPE, MH/PEW/LLDPE and MH/PEWM/LLDPE composites, and the LOI values are listed in Table 4. Clearly, the MH/LLDPE composite exhibits the highest LOI value (47.0%), but the LOI values of MH/LLDPE composites are reduced to different extents with the addition of PEW and PEWM. For the MH/PEWM/LLDPE and MH/PEW/LLDPE composites, the highest LOI value (46.0%) is obtained at 1 wt% of PEWM1, which is attributed to the improved dispersion of the MH particles. This is consistent with the CCT results. All samples exhibit extremely high LOI values (>43.0%), which indicates the satisfactory flame retardancy of MH. As shown in Figure 14C,D and Table 4, the peak smoke–production rate (PSPR) and THR values of the MH/LLDPE composites are 0.0095 m^2^/s and 2.13 m^2^/kg, respectively. Notably, PEWM1-1 exhibits the lowest PSPR (0.0092 m^2^/s) and TSP (1.48 m^2^/kg), which are 3.5% and 30.6% lower than that of the MH/LLDPE composites, respectively. Similar results are observed for PEWM1-2. However, the smoke–emission capacity of the MH/LLDPE composite is not inhibited with the addition of PEW1, PEW2 and PEWM2. In contrast, the addition of PEWM1 leads to the formation of a compact carbon layer during combustion, which might effectively decrease the release of smoke.

## 4. Conclusions

In this study, two MAH-grafted polyethylene waxes with almost the same grafting degree (3.62% and 3.60%) were prepared, both of which could improve the processability, fluidity and mechanical properties of highly filled MH/LLDPE composites. Compared to MH/LLDPE composites, the equilibrium torque of MH/PEWM/LLDPE composites with 1 wt% of PEWM1 was decreased by 20.0% and the MFI was increased by 667%. The tensile strength, elongation at break, and impact strength were increased by 21.29 MPa, 24.00% and 7.77 kJ/m^2^, respectively. The interfacial adhesion between the MH particles and LLDPE matrix was also enhanced. PEWM improved the dispersion of MH particles in the LLDPE matrix, which reduced the density of the molecular chain entanglement and enhanced the movement of molecular chains. However, PEW and PEWM may affect flame retardancy because of their low melting point and high combustibility in the early stage.

## Figures and Tables

**Table 1:** The formulations and names of MH/LLDPE composites.

No.	MH	LLDPE	PEW	PEW-g-MAH
MH/LLDPE	60	40	0	0
PEW1-1	60	39	1	0
PEW1-2	60	37	3	0
PEW1-3	60	35	5	0
PEW2-1	60	39	1	0
PEW2-2	60	37	3	0
PEW2-3	60	35	5	0
PEWM1-1	60	39	0	1
PEWM1-2	60	37	0	3
PEWM1-3	60	35	0	5
PEWM2-1	60	39	0	1
PEWM2-2	60	37	0	3
PEWM2-3	60	35	0	5

**Table 2:** Mechanical properties of all samples.

Samples	Tensile Strength (MPa)	Elongation at Break (%)	Impact Strength (kJ/m^2^)
MH/LLDPE	20.03 ± 1.10	23.42 ± 2.50	7.64 ± 0.78
PEW1-1	17.85 ± 0.25	18.88 ± 0.53	5.81 ± 0.33
PEW1-2	17.60 ± 0.61	16.69 ± 1.17	4.68 ± 0.17
PEW1-3	17.50 ± 0.72	15.68 ± 1.18	4.44 ± 0.19
PEW2-1	19.23 ± 1.06	20.38 ± 1.08	5.71 ± 0.19
PEW2-2	17.37 ± 0.99	16.53 ± 1.76	4.66 ± 0.24
PEW2-3	18.41 ± 1.02	18.76 ± 1.53	4.29 ± 0.10
PEWM1-1	21.29 ± 0.81	24.00 ± 1.54	7.77 ± 0.30
PEWM1-2	19.21 ± 0.88	19.71 ± 1.38	6.65 ± 0.11
PEWM1-3	20.16 ± 0.88	20.08 ± 0.72	4.94 ± 0.05
PEWM2-1	19.56 ± 0.83	21.40 ± 0.68	7.25 ± 0.15
PEWM2-2	19.25 ± 2.06	22.61 ± 1.32	5.52 ± 0.52
PEWM2-3	19.13 ± 0.80	25.80 ± 1.95	4.96 ± 0.28

**Table 3:** Some TGA characteristic parameters of the composites.

Samples	Temperature (°C)		
	T_5%_	T_max_	Char Yield (%)
MH/LLDPE	403.1	477.0	41.9
PEW1-1	396.4	475.3	41.4
PEW2-1	395.3	476.1	41.2
PEWM1-1	403.4	477.6	42.2
PEWM1-3	400.6	476.5	39.4
PEWM2-1	397.7	476.8	41.3
PEWM2-3	362.3	476.3	41.0

**Table 4:** Cone data for composites.

Samples	LOI (%)	T_ign_ (s)	PHRR (kW/m^2^)	THR (MJ/m^2^)	PSPR (m^2^/s)	TSP (m^2^/kg)	FPI (10^−2^)	AMLR (g/s)
MH/LLDPE	47.0	160	123.75	58.43	0.0095	2.13	129.29	1.52
PEW1-1	45.0	138	158.53	68.54	0.012	2.78	87.05	2.04
PEWM1-1	46.0	141	133.65	65.68	0.0092	1.48	105.50	1.90
PEWM1-3	44.0	121	148.10	68.49	0.010	2.00	81.70	1.60
PEW2-1	44.7	136	179.55	69.85	0.011	2.86	75.75	2.18
PEWM2-1	45.8	138	175.11	67.28	0.0094	2.53	78.81	2.00
PEWM2-3	43.9	126	177.83	70.66	0.012	3.38	70.86	2.29

## Data Availability

Not applicable.
